# Strategic inhibition of distractors with visual working memory contents after involuntary attention capture

**DOI:** 10.1038/s41598-017-16305-5

**Published:** 2017-11-24

**Authors:** Jiachen Lu, Lili Tian, Jiafeng Zhang, Jing Wang, Chaoxiong Ye, Qiang Liu

**Affiliations:** 1 Research Center of Brain and Cognitive Neuroscience, Liaoning Normal University, Dalian, 116029 China; 2 Department of Psychology, University of Jyväskylä, Jyväskylä, 40014 Finland; 3 Key Laboratory for NeuroInformation of Ministry of Education, University of Electronic Science and Technology of China, Chengdu, 610054 China

## Abstract

Previous research has suggested that visual working memory (VWM) contents had a guiding effect on selective attention, and once participants realized that the distractors shared the same information with VWM contents in the search task, they would strategically inhibit the potential distractors with VWM contents. However, previous behavioral studies could not reveal the way how distractors with VWM contents are inhibited strategically. By employing the eye-tracking technique and a dual-task paradigm, we manipulated the probability of memory items occurring as distractors to explore this issue. Consistent with previous behavioral studies, the results showed that the inhibitory effect occurred only in the high-probability condition, while the guiding effect emerged in the low-probability condition. More importantly, the eye-movement results indicated that in the high-probability condition, once few (even one) distractors with VWM contents were captured at first, all the remaining distractors with VWM contents would be rejected as a whole. However, in the low-probability condition, attention could be captured by the majority of distractors with VWM contents. These results suggested that the guiding effect of VWM contents on attention is involuntary in the early stage of visual search. After the completion of this involuntary stage, the guiding effect of task-irrelevant VWM contents on attention could be strategically controlled.

## Introduction

The working memory system plays an important role in human cognition, as it enables our brains to retain and manipulate external information temporarily^1–4^. Recent studies have focused on exploring the interactions of visual working memory (VWM) and selective attention, with results showing that VWM and selective attention involve several overlapping brain regions, including the right frontal-parietal cortex (rFPC), the right occipital cortex (rOLC) and the bilateral insula^5–7^. The overlapping anatomical structures of VWM and selective attention suggest that there might also be a functional coupling between them. Therefore, an understanding the interaction of VWM and selective attention is of great importance for the study of human cognition.

To disclose the mechanism of this interaction, Desimone and Duncan^8^ proposed the biased competition model, which, for the first time, elucidated the role of VWM content in attentional guidance. According to the biased competition model, the load of objects that are presented simultaneously in the visual field is usually beyond the processing capacity of the human brain. Consequently, these objects would compete against each other to win more cognitive resources in order to achieve further processing. Such competition was assumed to be influenced by the VWM content in a top-down manner^9^. Specifically, representations matching the VWM content would be reinforced by attracting more attention. The biased competition model was supported by a behavioral study by Downing^10^. In Downing’s experiment, a facial picture (the sample) was presented to participants for memorizing. After the sample had disappeared from view, another two pictures (one matching the sample and the other novel) were randomly presented on each side of the screen as distractors. Participants were required to perform a discrimination task to judge the orientation of a bracket appearing at the location of either one of the two pictures. At the end of each trial, a single face was presented and participants were required to report whether or not it matched the one held in their VWM. The results showed that reaction time (RT) was significantly shortened when the brackets occurred at the location of the memory-matched pictures rather than the non-memory-matched ones. Based on these results, the study claimed that memory-matched stimuli gained more advantages in capturing attention and concluded that VWM content exerted a guiding effect on selective attention.

Since Downing’s study^10^, researchers began to pay more attention to the guiding effect of the content of VWM on selective attention, and subsequently conducted a series of empirical research around this topic^11–16^. These studies adopted a mixed search task^17^, in which the participants needed to perform two types of tasks, namely, a change detection task and a visual search task. The search task was inserted during the memory retention phase of the change detection task. In the experiment, the relationship between a memory item and its subsequent search array was designed as three separate conditions: (1) valid matching condition: matching between features of the memory item and the search target; (2) invalid matching condition: matching between features of the memory item and the distractor; and (3) neutral condition: no occurrence of any feature of the memory item in the search array. The results showed that, compared with the neutral condition, the RT for searching was faster for the valid matching condition and slower for the invalid matching condition. These results provided further supporting evidence of the guiding effect. More importantly, by adopting the event-related potentials (ERP) technique, Kumar *et al*.^13^ found that the amplitude of N2pc was larger in an ipsilateral invalid matching condition (the search target was presented in the same visual field as the distractor for memory content), compared with the neutral condition and a contralateral invalid matching condition. According to previous research, N2pc has been regarded as an index of attentional processing^18^. The ERP results indicated that items sharing the same feature with the memory items would automatically capture attention in the search array.

Although many studies have provided evidence for the biased competition model by articulating that VWM has a guiding effect on the allocation of attention, the reliability of this model has been challenged by recent research proposing that VWM content does not always exert a guiding effect on the allocation of attention, but rather that such a guiding effect is mediated by cognitive strategy^19–24^. In Woodman and Luck’s experiment^21^, participants were told to keep the color information for one item in mind, which served as the contents of their VWM. Participants were also informed before the experiment that the color held in their memory would occur only in the distractors of the search task. The study found that the RT of searching was faster in the invalid matching condition compared with the neutral condition. For the interpretation of the results, Woodman and Luck^21^ proposed that once participants were aware of the distractive role of VWM content in the search task, they would strategically exclude it into a rejection template to inhibit all potential memory-matched distractors. Since attention was not allocated to those distractors, the searching efficiency was consequently enhanced^20–23^.

A synthesis of findings from previous studies that are inconsistent with the biased competition model revealed some commonalities among them. The experiments in those studies usually contained several memory-matched distractors. Multiple distractors, coupled with the difficulty of the search task, made it difficult for participants to make fast judgments, which resulted in relatively longer RTs (more than 1000 ms on average)^21–23^. Thus, it could be speculated that this elongated process likely involves multiple cognitive processes. Previous findings showing that N2pc^25,26^ was modulated by VWM allowed for the prediction that the guiding effect of VWM content on attention would occur at an early period after stimulus onset. Therefore, we presumed that the faster RT in the invalid matching condition might result from the following three possibilities: (1) Participants strategically inhibited all the memory-matched distractors and allocated no attention to any distractors, which consequently enhanced the searching efficiency and shortened the searching time. In the current study, we named this possibility the *inhibiting hypothesis*. (2) When a memory-matched distractor captured the participants’ attention for the first time, participants would become aware of the distractive role of VWM content in the visual search. As a result, all memory-matched distractors would be rejected as a whole, which consequently sped up the searching time. This possibility was named the *guiding-inhibiting hypothesis*. (3) Memory-matched distractors would preferentially capture participants’ attention, and then these items would automatically be recognized as distractors with the clue of captured signals. As memory-matched distractors required no further judgment of the task-related features, this would shorten the processing time for memory-matched distractors and, therefore, enhance the searching efficiency. This possibility is referred to as the *guiding hypothesis*.

Therefore, it can be concluded that the results claiming the rejection effect did not necessarily exclude the guiding effect of VWM content on attention. However, behavioral measures (RT) are a result of multiple cognitive processes, which are limited per se in distinguishing different types of processing mechanisms. Being aware of this limitation, the present study aimed to examine the above three hypotheses by adopting the eye-tracking technique, which possesses advantages over behavioral measurement techniques in observing the distribution of attention across both spatial and temporal dimensions.

In the current study, participants were at first required to memorize a colored shape and then to perform a visual search task. In the visual search, the participants needed to search for one target item among six items. A square frame with a gap was inserted at the central position of the colored shape to serve as the searching item. Two colored shapes were randomly selected from the stimulus pool (120 colored shapes) in each trial. Each of the three items in the search array shared the same type of colored shapes. We defined the target and the other two distractors sharing the same colored shape as the target-analogue items, and another three distractors sharing the other colored shape as the non-target-analogue items. For the non-target-analogue items, the colored shapes were likely to be either identical with the previously memorized colored shape (memory distraction trial) or different from the memorized colored shape (control distraction trial). By manipulating the occurrence probability of the VWM content as distractors, the current study aimed to explore the effect of VWM content on the allocation of attention. It was predicted that in the high-probability condition, an inhibitory effect was likely to be observed, with a shorter searching RT in the memory distraction trials than in the control distraction trials, while in the low-probability condition, a guiding effect on attention was likely to be observed with a longer searching RT in the memory distraction trials than in the control distraction trials.

In terms of the eye-movement results, it was presumed that three different results were likely to occur in the high-probability condition. (1) According to the *inhibiting hypothesis*, the participants would effectively inhibit all memory-matched distractors. Therefore, it was predicted that the number of non-target-analogue items that the participants would scan would be significantly lower in the memory distraction trials than in the control distraction trials. In addition, the probability of the first scanned item being a non-target-analogue item would be significantly lower in the memory distraction trials than in the control distraction trials. (2) According to the *guiding-inhibition hypothesis*, one of the memory-matched distractors would first capture the participants’ attention, and then the remaining memory-matched distractors would be inhibited due to the awareness of the distractive role of VWM content. Consequently, the number of scanned non-target-analogue items would be significantly lower in the memory distraction trials than in the control distraction trials. Meanwhile, the probability of the first scanned item being a non-target-analogue item would be significantly higher in the memory distraction trials than in the control distraction trials. (3) According to the *guiding hypothesis*, memory-matched distractors would preferentially capture the participants’ attention and then automatically be recognized as distractors due to the clue of captured signals. Thus, it was predicted that the number of scanned no-target-analogue items would be significantly higher in the memory distraction trials than in the control distraction trials. Moreover, in scan order, the probability of the first three scanned items being non-target-analogue items would be significantly higher in the memory distraction trials than in the controlled distraction trials, while the gaze duration for a scanned non-target-analogue item would be shorter in the memory distraction trials than in the control distraction trials.

Since the rejection effect would not occur in the low-probability condition, memory-matched distractors would capture the participants’ attention. Regarding the eye-movement measures, the number of scanned non-target-analogue items would be significantly higher in the memory distraction trials than in the control distraction trials.

## Methods

### Participants

A total of 30 participants were recruited from Liaoning Normal University (12 males and 18 females, aged between 18 and 22 years, M = 20.08, SD = 1.42). Participants were divided into two groups, with 15 participants being randomly assigned to each group. All participants in the experiment had normal or corrected-to-normal visual acuity and were right-handed. All participants received compensation for their involvement in the experiment. Written informed consent was provided by each participant prior to the experiment. The study conformed to the Declaration of Helsinki and was approved by the ethics committee of Liaoning Normal University.

### Apparatus and stimulus

Participants were seated in a sound-proof, dark room at a distance of 70 cm from a 17-in screen. The stimuli were presented on a screen with a white background. All memory items were 1.8° × 1.8° colored shapes. The stimulus pool consisted of 60 original, colored shapes (six colors: blue, gold, yellow, light green, dark green, pink; ten shapes: bubble, right triangle, isosceles triangle, regular triangle, trapezoid, oval, incomplete circle, diamond, parallelogram, regular parallelogram). The sixty original, colored shapes were rotated 180° to get a total of 120 colored shapes.

**Figure 1:**
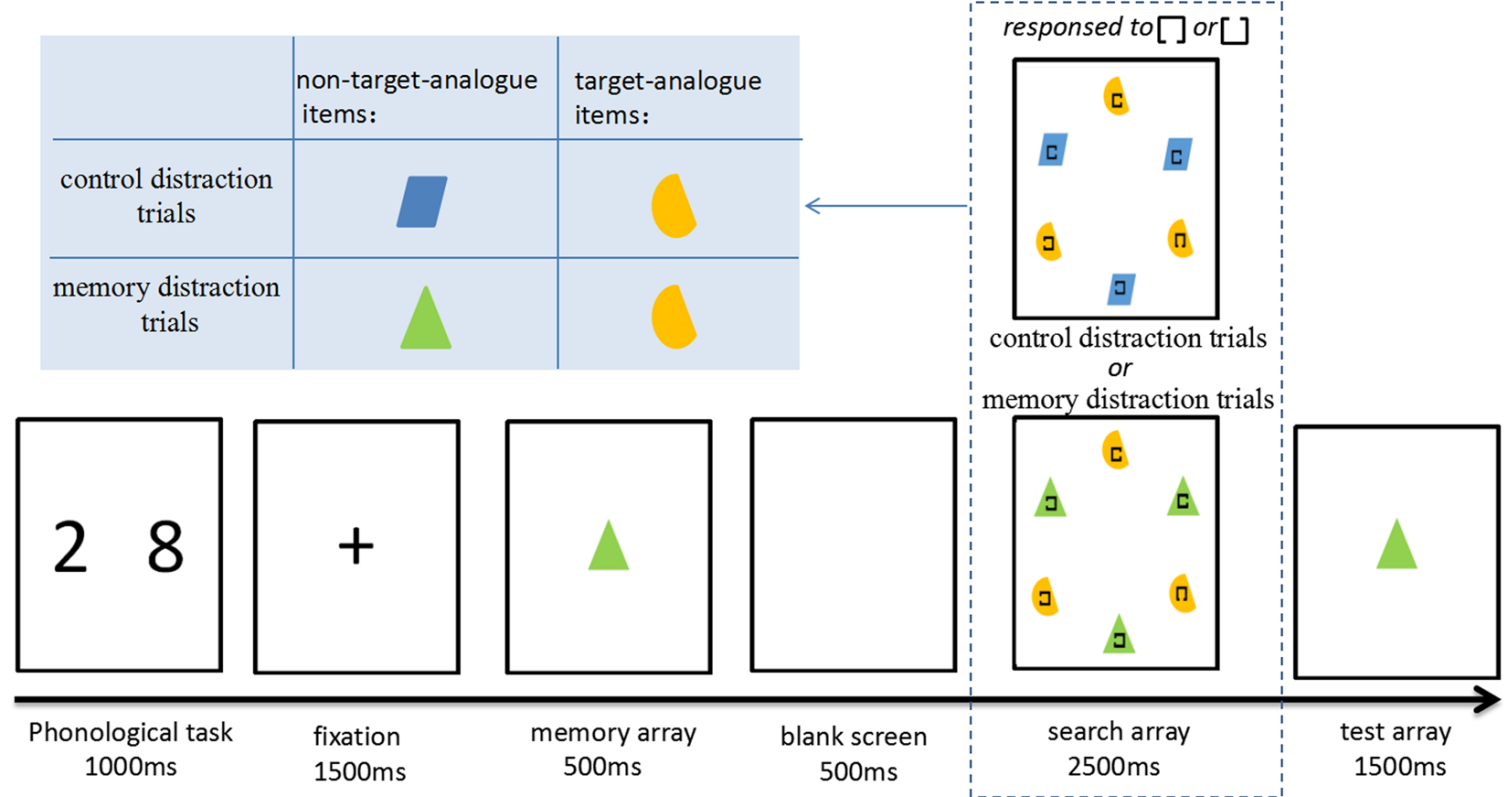
Trial structure of the dual-task paradigm. In this example, there are two kinds of items (non-target-analogue items and target-analogue items) in the search array. The non-target-analogue items sharing the same colored shape with the memory item and would occur only in the memory distraction trials but not in the control distraction trials. The target would appear only in the target-analogue items. The paradigm consisted of two different types of trials.

The search array (Fig. 1) consisted of six items that were evenly distributed throughout a virtual disk with an angle of 9° and corresponding clockwise to 2, 4, 6, 8, 10 and 12, or 1, 3, 5, 7, 9 and 11. The distance from each item to the central position of the screen was 4.6° and the minimum distance between items was 1.6° to ensure that no interference occurred among the items. A square frame with a 0.2° gap was inserted at the 0.6° central position of the colored shapes to serve as a searching item. The search array included one target with either an up or down opening-direction square-frame and five distractors with either left or right opening-direction square-frames. Two types of items were designed in the search array, namely, non-target-analogue items and target-analogue items. The non-target-analogue items included three distractors sharing the same colored shape, and the target-analogue items included a target and two distractors sharing another colored shape.

Experiment Builder was used for the experimental programming and EyeLink 1000 plus (SR Research, Ontario, Canada) was employed to collect the eye movement data. The sampling frequency was 1000 Hz. Viewing was monocular and the eye movement data were collected from the right eye. The EyeLink system used an ethernet link between the eye tracker and the display computers so that the real-time gaze position data could be displayed. Two computer monitors were employed in this experiment, with one of them being used to display experimental materials to the participants, and the other being used to display real-time feedback of the participants’ eye movements to the experimenter. The participants rested their heads on a chin rest to minimize head movements during breaks in the experiment.

### Experimental design

The experiment employed a 2 × 2 mixed design, with the type of trials (memory distraction trials, control distraction trials) as a within-subject variable and the occurrence probability of the memory distraction trials in the visual search task (low probability condition [20%] vs. high probability condition [80%]) as a between-subject variable. The colored shape of the non-target-analogue items was consistent with the previous memory items in the memory distraction trials but not in the control distraction trials. Furthermore, neither the color feature nor the shape feature of the target-analogue items was consistent with the previous memory item.

### Experiment procedure

As shown in Fig. 1, each trial started with the presentation of two randomly selected digits (ranging from 2 to 9, the size of each digit was 0.8° × 1.2°) for 1000 ms. These two digits were presented in the left and right visual field, with a visual angle distance of 1.8° from the center of the screen. Participants were instructed to repeat the digits consecutively at a rate of 4 digits per second at the beginning of each trial to the end of the visual search. The reading task was designed to encourage visual coding processing and to prevent phonological coding, which could have exerted influence on the experiment performance^27^. After 1500 ms of a blank screen, a memory array was presented for a duration of 500 ms. Each memory array included a colored shape, which needed to be memorized by the participants. Following a 500 ms delay after the memory item disappeared, a search array was presented for 2500 ms. Within the search array, the participants were required to judge the opening direction (up or down) of the square-frame-embedded items and to make a response by pressing a key (“5” for “up”; “2” for down). The participants were informed before the experiment that the colored shape of the target item was always different from that in the memory array. They were also informed about the real probability of the memory distraction trials in the visual search task. In the test array, which followed the search array, an item was presented in the center of the screen. The participants were required to judge whether the test item was the same as the memory item. To make sure that the participants memorized both the color and shape feature of the memory item, in half of the trials, the test item was designed to share the same color and shape as the memory item (press “Y” key); in one-sixth of the trials, it was designed to share only the same color; in one-sixth of the trials, it was designed to share only the same shape; and in the remaining third of the trials, it was designed to share neither the same color nor the same shape (press “N” key in the three conditions).

All participants were required to finish a total of 120 trials. A short break was set between every 40 trials (at least 30 s). The memory distraction trials appeared 96 times in the high-probability condition and 24 times in the low-probability condition. The memory distraction trials and the control distraction trials occurred alternately and were randomized. The participants were provided with 15 practice trials to become familiar with the procedure before the formal experiment.

### Data analysis

#### Behavioral results and analysis

We first measured the memory accuracy of the test array. In the high-probability condition, the accuracy results of the memory distraction trials and the control distraction trials were 0.88 (SD = 0.07) and 0.83 (SD = 0.08), respectively. In the low-probability condition, the accuracy results of the memory distraction trials and the control distraction trials were 0.86 (SD = 0.07) and 0.80 (SD = 0.05), respectively. A 2 × 2 mixed design analysis of variance (ANOVA) was conducted to analyze the memory accuracy of the test array. No significant main effect of probability was found, *F*(1,28) = 0.29, *p* > 0.05, *η* ^2^ = 0.04. A significant main effect of type of trials was observed, *F*(1,28) = 11.66, *p* < 0.05, *η* ^2^ = 0.29. No significant interaction effect was found between probability and type of trials. The behavioral results showed that items with memory information in the search array facilitated memory consolidation and therefore, improved the accuracy of the memory detection task.

**Figure 2:**
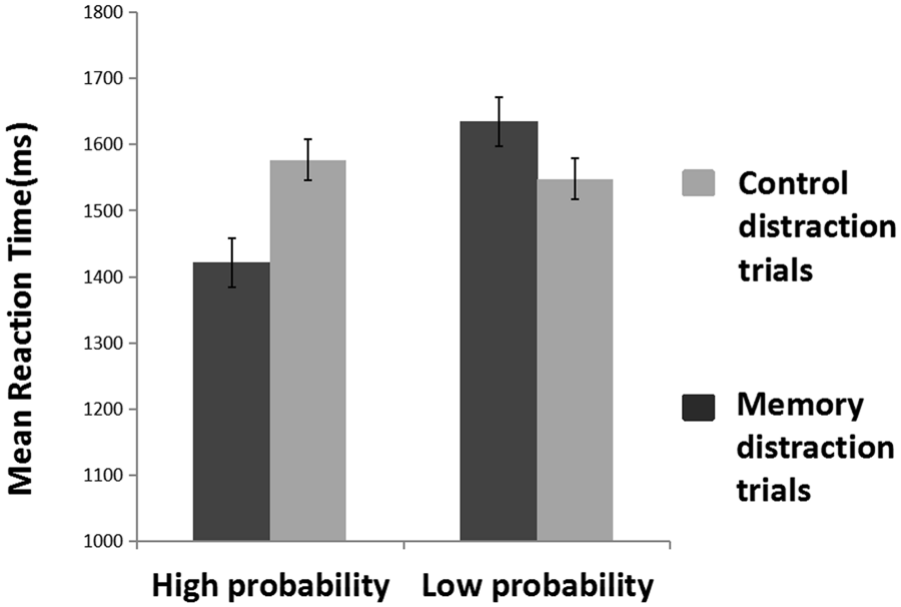
The results of the reaction times. Error bars are SEM.

Figure 2 presents the overall RT of the trials with correct responses in the memory detection task. A 2 × 2 mixed design analysis of variance (ANOVA) was conducted with the occurrence probability of the memory distraction trials (high-probability vs. low-probability) as the between-subject variable and the type of trials (memory distraction vs. control distraction) as the within-subject variable. The statistical results showed a significant main effect of probability, *F*(1,28) = 7.59, *p* = 0.01, *η* ^2^ = 0.21. The main effect of type of trials was also significant, *F*(1,28) = 4.7, *p* < 0.05, *η* ^2^ = 0.144. The interaction effect between the probability and type of trials was also significant, *F*(1,28) = 35.20, *p* < 0.001, *η* ^2^ = 0.56. Simple effect analysis further revealed that in the high-probability condition, the mean RT of the memory distraction trials was significantly shorter than that of the control distraction trials (1440 ms vs. 1606 ms), *F*(1,14) = 14.44, *p* = 0.001, *η* ^2^ = 0.34. In the low-probability condition, the mean RT was significantly longer for the memory distraction trials than for the control distraction trials (1620 ms vs. 1550 ms), *F*(1,14) = 5.70, *p* < 0.05, *η* ^2^ = 0.17.

The behavioral results indicated that in the low-probability condition, the memory-matched distractors were more likely to capture the participants’ attention, which therefore led to a longer searching RT. In the high-probability condition, the memory-matched distractors were more likely to be inhibited and the searching RT was consequently shortened.

#### Eye movement results and analysis

**Figure 3:**
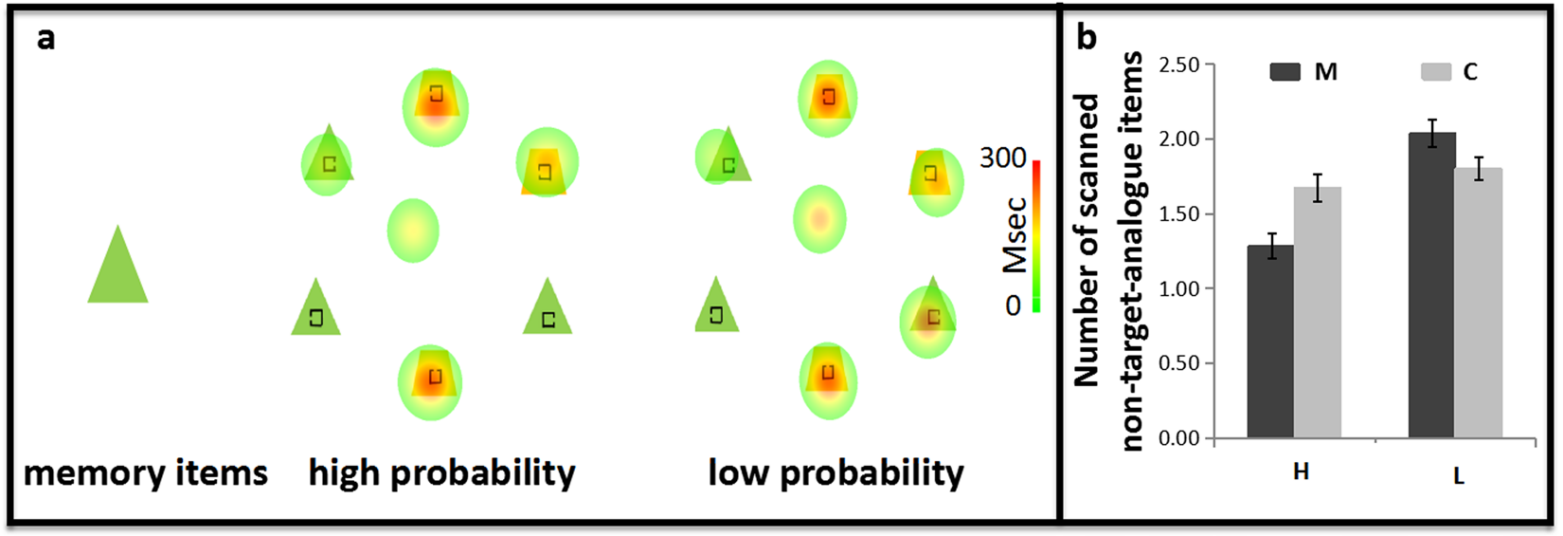
The results of spatial distribution of the fixations. (**a**) The heat maps of the spatial distribution of the fixations are plotted separately for the high and low probability conditions. The colors represent the length of fixation duration. (**b**) The average number of non-target-analogue items that were scanned are plotted separately for the high- and low- probability conditions. M: Memory distraction trials; C: Control distraction trials; L: Low-probability condition; H: High-probability condition. Error bars are SEM.

As shown in Fig. 3a, we defined the region of interest (ROI) as a circle with 2° angle that was centered on each item. Thus, there were six ROIs in total, with each item located at the center of its ROI. Fixations located at ROIs would be further analyzed. The incorrect response trials of the memory detection task were excluded in the eye-movement data analysis.

#### Spatial distribution of fixations

Figure 3a shows two heat maps from one trial each of the high- and low- probability conditions. Participants in the high-probability condition tended to reject the rest of the two memory-matched distractors once they had become aware of the distractive role of the memory contents in whichever of the three items and then continued to search the target from the three target-analogue items. Participants in the low-probability condition would pay attention to all the three memory-matched distractors and reject none of them.

Figure 3b presents the average number of non-target-analogue items that were detected in each condition. A 2 (probability) × 2 (type of trials) repeated measures ANOVA analysis revealed a significant main effect of *probability*, *F*(1,28) = 33.47, *p* < 0.001, *η* ^2^ = 0.55. The main effect of *type of trials* was not significant, *F*(1,28) = 0.62, *p* > 0.1, *η* ^2^ = 0.02. The interaction effect was significant *F*(1,28) = 10.59, *p* < 0.01, *η* ^2^ = 0.24. Simple effect analysis showed that in the high-probability condition, the number was significantly lower in the memory distraction trials than in the control distraction trials, *F*(1,14) = 9.47, *p* < 0.01, *η* ^2^ = 0.25; while in the low-probability condition, the number was higher in the memory distraction trials that in the control distraction trials, *F*(1,14) = 3.95, *p* = 0.057, *η* ^2^ = 0.14. In addition, in the memory distraction trials, the number was significantly lower in the high-probability condition than in the low-probability condition, *F*(1,28) = 36.70, *p* < 0.001, *η* ^2^ = 0.57. On the control distraction trials, the number in the high-probability and the low-probability conditions showed no significant difference, *F*(1,28) = 1.16, *p* > 0.1, *η* ^2^ = 0.04.

The above results indicated that in the high-probability condition, once a few distractors (even one) with memory content had captured the participants’ attention successfully, all distractors with VWM content would be rejected as a whole. The participants would continue to search the target from the rest of the target-analogue items. Nevertheless, in the low-probability condition, the participants tended to be attracted to any of the distractors with VWM content and then would search the target from the rest of the target-analogue items. Although the number of the detected distractors in the low-probability condition was 2.03 rather than 3, it was still unconvincing to claim that this result was caused by rejecting distractors with VWM content. The reason why the fixation failed to fall on the third distractor with VWM content may be attributed to the fact that the elimination of distractors with VWM content enhanced the proportion of target-analogue items in the total number of search items. The quantitative advantage of the target-analogue items made it easier for the target-analogue items to gain attention. In summary, in the high-probability condition, VWM content influenced attention in a half-flexible manner, which initially guided but then inhibited attention, while in the low-probability condition, VWM content exerted a guiding effect only on the allocation of attention, and no inhibitory effect was observed.

#### Scan Order Analysis

**Figure 4:**
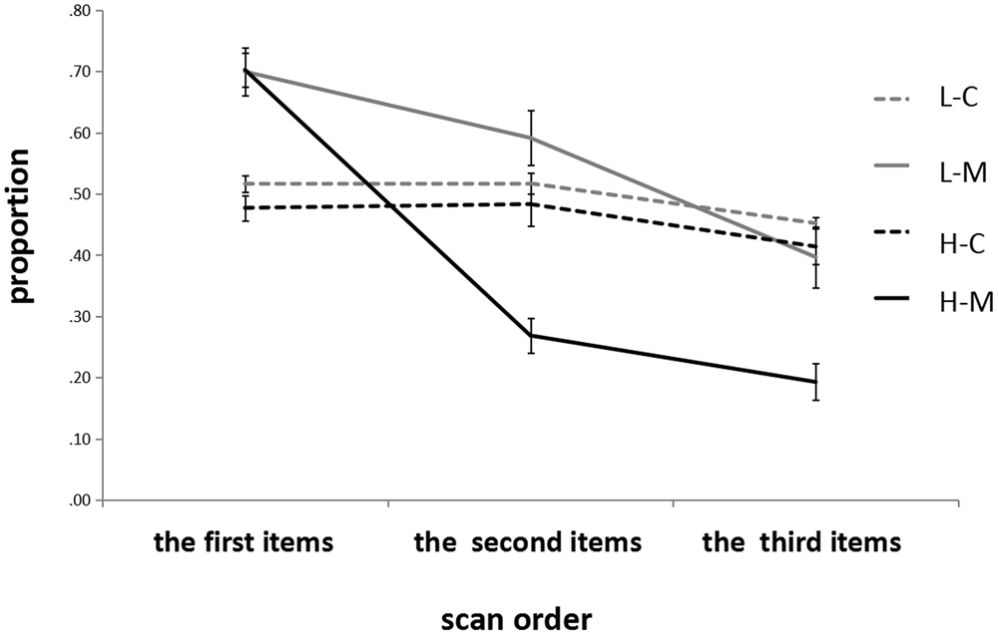
The possibility that the non-target-analogue items are scanned at first three scanned items. L-C: Control distraction trials in the low-probability condition; L-M: Memory distraction trials in the low-probability condition; H-C: Control distraction trials in the high-probability condition; H-M: Memory distraction trials in the high-probability condition. Error bars are SEM.

As shown in Fig. 3b, the number of non-target-analogue items that were scanned was lower in the high-probability condition than in the low-probability condition, which indicated the occurrence of an inhibitory effect in the high-probability condition rather than the in the low-probability condition. To investigate the onset time of the inhibitory effect, the scanned items were analyzed in a proper sequence. Since the number of memory-matched distractors in the search array was 3, and the average number of scanned items was 3.34 (SD = 0.63), the present statistical analysis was conducted concerning only the first three scanned items. As shown in Fig. 4, the first three scanned items of each participant are presented. The percentage of the non-target-analogue items in each sequence was analyzed. A 2 (*probability*) × 2 (*type of trials*) × *3* (*searching order*) repeated measures ANOVA was performed to analyze the percentage of the non-target-analogue. The results showed that the main effect of *probability* was significant, *F*(1,28) = 26.63, *p* < 0.001, *η* ^2^ = 0.49; the main effect of *type of trials* was not significant, *F*(1,28) = 0.01, *p* > 0.1, *η* ^2^ = 0.00; and that the main effect of order was significant, *F*(1,27) = 61.76, *p* < 0.001, *η* ^2^ = 0.82. The interaction effect of *probability*, *type of trials* and *order of items* was significant, *F*(1,27) = 6.28, *p* < 0.01, *η* ^2^ = 0.32. The subsequent analysis showed that in terms of the first item scanned, the main effect of *probability* was not significant, *F*(1,28) = 0.42, *p* > 0.1, *η* ^2^ = 0.03; the main effect of *type of trials* was significant, *F*(1,28) = 63.03, *p* < 0.01, *η* ^2^ = 0.55; and the interaction effect was not significant, *F*(1,28) = 0.68, *p* > 0.1, *η* ^2^ = 0.01. This result indicated that when the memory items appeared as distractors in the visual search task, the memory-matched distractors would attract more attentional resources compared with the non-memory-matched distractors in spite of a high or low occurrence probability. In terms of the second item scanned, the main effect of *probability* was significant, *F*(1,28) = 27.77, *p* *<* 0.01, *η* ^2^ = 0.50; the main effect of *type of trials* was significant, *F*(1,28) = 4.82, *p* < 0.05, *η* ^2^ = 0.15; and the interaction effect was significant, *F*(1,28) = 20.41, *p* < 0.01, *η* ^2^ = 0.42. Simple effect analysis showed that in the high-probability condition, the percentage of searched non-target-analogue items was significantly lower in the memory distraction trials than in the control distraction trials, *F*(1,14) = 10.30, *p* < 0.01, *η* ^2^ = 0.27. In the low-probability condition, the percentage of non-target-analogue items in the memory distraction trials and the control distraction trials showed no significant difference, *F*(1,14) = 2.44, *p* = 0.13, *η* ^2^ = 0.34. In terms of the third item scanned, the main effect of *probability* was significant, *F*(1,28) = 13.87, *p* = 0.01, *η* ^2^ = 0.33; the main effect of *type of trials* was significant, *F*(1,28) = 17.83, *p* < 0.01, *η* ^2^ = 0.39; and the interaction effect was also significant, *F*(1,28) = 6.42, *p* < 0.05, *η* ^2^ = 0.19. Simple effect analysis showed that in the high-probability condition, the percentage of non-target-analogue items was significantly lower in the memory distraction trials than in the low-probability condition, *F*(1,14) = 28.32, *p* < 0.01, *η* ^2^ = 0.50. In the low-probability condition, the percentage of non-target-analogue items in the memory distraction trials and the control distraction trials showed no significant difference, *F*(1,14) = 1.21, *p* > 0.1, *η* ^2^ = 0.04. Through analyzing the scan order of the three items, it was revealed that in the high-probability condition, after attention was captured by the first memory-matched distractor, the participants did not continue to scan the remaining two memory-matched items. In other words, the inhibitory effect appeared in these two memory-matched distractors. In the low-probability condition, after attention was captured by the first memory-matched distractor, the remaining two memory-matched distractors also succeeded in capturing the participants’ attention.

To investigate whether the guiding effect of VWM content on attention would facilitate the speed of fixation shifting to the memory-matched distractors, the current study conducted a 2 (*probability*) × 2 (*type of trials*) repeated measures ANOVA in terms of *mean entry time*, which was the time required for the first fixation to occur on the items (as shown in 5a). The results showed that the main effect of *probability* was not significant, *F*(1,28) = 0.50, *p* > 0.1, *η* ^2^ = 0.2; the main effect of *type of trials* was not significant, *F*(1,28) = 0.50, *p* > 0.1, *η* ^2^ = 0.2; and the interaction effect was not significant, *F*(1,28) = 1.94, *p* > 0.1, *η* ^2^ = 0.65. The guiding effect did not occur in *mean entry time*.

**Figure 5:**
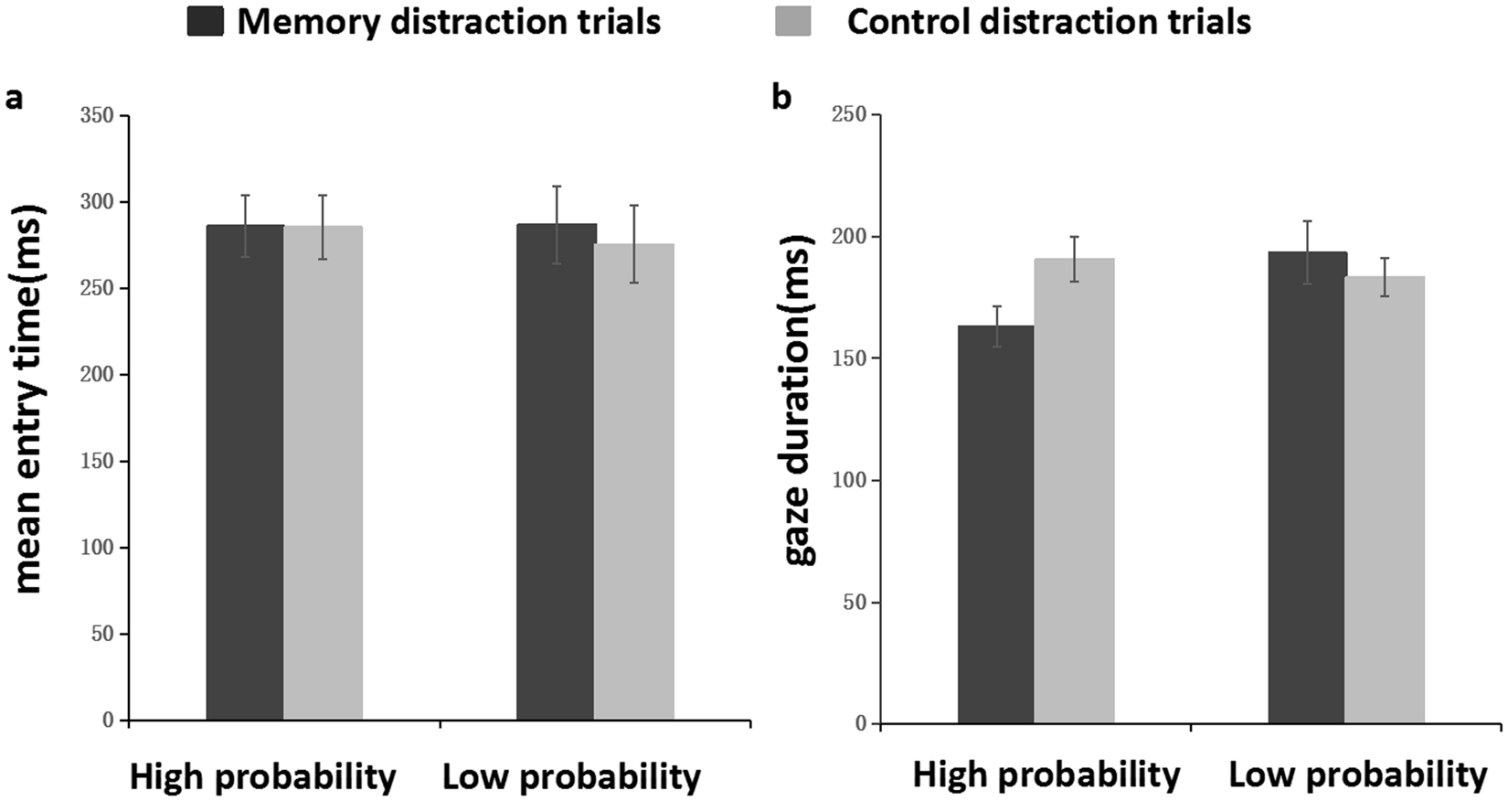
The results of (**a**) the mean entry time of the first scanned Items and (**b**) the gaze duration of first scanned item. The results are plotted separately on both memory distractive and controlled distractive trials for the high and low probability conditions. Error bars are SEM.

As shown in Fig. 5b,a 2 (probability) × 2 (type of trials) repeated measures ANOVA was conducted concerning the gaze duration of the first scanned item. The results showed that the main effect of *probability* was not significant, *F*(1,28) = 0.90, *p* > 0.1, *η* ^2^ = 0.03; the main effect of type of trials was not significant, *F*(1,28) = 2.01, *p* > 0.05, *η* ^2^ = 0.07; and that the interaction effect was significant, *F*(1,28) = 8.84, *p* < 0.01, *η* ^2^ = 0.24. Simple effect analysis showed that in the high-probability condition, the gaze duration was significantly shorter in the memory distraction trials than in the control distraction trials, *F*(1,14) = 5.01, *p* < 0.05, *η* ^2^ = 0.15. In the low-probability condition, the gaze duration in the memory distraction trials and the control distraction trials showed no significant difference, *F*(1,14) = 0.43, *p* > 0.1, *η* ^2^ = 0.02. Taken together, the results indicated that in the high-probability condition, after being attracted by the first memory-matched distractor, the participants were very likely to stop discriminating the opening direction of the square-frame that was embedded in the rectangle and directly shift their fixation away from the memory-matched distractors.

## Discussion

The current study investigated the role of VWM content in rejecting distractors when participants were clearly informed of the distractive role of the memory-matched items in the visual search task. The searching RT results indicated that VWM content exerted an inhibitory effect in the high-probability condition and an attentional capture effect in the low-probability condition. The eye-movement results allowed for further examination of how searching RT results varied in the different cognitive stages. The results showed that when the memory distraction trials appeared in the visual search task with a high probability, VWM content would capture attention at an early stage of the visual search^28–33^. This result failed to support the direct inhibitory mechanism based on the inhibiting hypothesis. Furthermore, once a few (even one item) distractors with VWM content successfully captured attention, all the remaining distractors with VWM content would be wholly rejected. Then, the participants would continue to search the target item from the rest of the target-analogue ones. This finding meshed well with the guiding-inhibiting hypothesis, which claimed whole rejection preceded by attentional capture.

Actually, the guiding-inhibiting hypothesis provided partial explanation and insight for the current debate about whether the guiding effect of VWM content on attention is involuntary or is under strategic control. The guiding-inhibiting hypothesis suggests that the guiding effect of VWM content on attention is involuntary at an early stage of the visual search. On the other hand, the hypothesis suggests that attention would move away from distractors upon the identification of a few memory-matched distractors, which is consistent with the view of the strategic control. Moreover, it would be inferred that the cognitive control of the task-irrelevant VWM information demanded sufficient time to come into effect^16,28,34^. This inference explained why the strategic control held true only when the visual search task took a longer time to perform. The inhibitory mechanism in the current study can be interpreted by the theory of templates for rejection proposed by Woodman and Luck^21^. According to Woodman and Luck, the VWM information would form a template for rejection on the condition that the participants were clearly informed of the distractive role of the memory-matched items. However, Woodman and Luck^21^ did not elucidate the details of how the rejection template modulated the allocation of attention when several items with memory content occurred simultaneously. The current study suggests that the rejection template would be initiated only when a few (even one item) memory-matched distractors would capture the participants’ attention and make them aware of the occurrence of the memory-matched distractors. Furthermore, the current study also suggests that the speed of the searching response would certainly be reduced when only one distractor with VWM content occurred in the search array. This seems to contradict the results from Woodman and Luck’s^21^ study. In their Experiment 3, a faster response in the memory distraction trials rather than in the control distraction trials was observed when a memory-matched distractor was presented. One possible explanation could be that in Woodman and Luck’s^21^ experiment, the search array contained several items (six items), which made it difficult for the participants to identify the target. In the task, the participants were required to distinguish and process each item one by one to meet the target item. In this difficult situation, the target would not be obtained until a large number of items were distinguished and recognized in sequence. Although the memory-matched distractor captured the participants’ attention at first and was processed with priority, the average number of scanned items was not affected. As the current results demonstrated, the participants could automatically and rapidly recognize the memory-matched items as distractors/non-target based on the signal of attentional capture, which would require no further judgment of its task-related features, and thus, attention would directly shift to the next item. This processing manner shortened the processing time for the memory-matched distractors and consequently enhanced the searching efficiency.

However, previous studies have found that when the occurrence rate of distractors was high, the participants could directly inhibit the distractors in a proactive way without being caught by the attention^24,35^. This is not consistent with the results of the present study. A possible explanation is that in these studies, the target was presented in the center of the screen only, and the distractors were always presented in a fixed position on both sides of the target. The participants could pre-suppress the location of the distractors to avoid interference. However, the target position was not fixed in the present study. Although the participants knew that distractors with VWM content would appear with a high probability (80%), the target items would appear randomly at one of six locations. The participants could not filter the memory-matched distractors by pre-suppression of certain locations. Therefore, VWM content would capture attention at an early stage of the visual search.

Regarding accuracy, the memory distraction trials had significantly higher memory accuracy than the control distraction trials. According to the interpretation of Woodman and Luck^21^, since the presentation duration of the search array was 2500 ms, after finishing the visual search task, the participants had sufficient time to shift their attention to the memory-matched distractors for consolidation (in the memory distraction trials, the mean RT was 1440 ms in the low-probability condition and 1606 ms in the high-probability condition), which reinforced the VWM representations and resulted in a higher memory accuracy in the memory distraction trials.

## Conclusion

The current study suggested that the guiding effect of VWM content on attention is involuntary at an early stage of visual search. In addition, the guiding effect of task-irrelevant VWM content on attention could be strategically controlled. Furthermore, the cognitive control of task-irrelevant VWM content demands sufficient time to come into effect.
